# Identification of the Similarities and Differences of Molecular Networks Associated With Fear Memory Formation, Extinction, and Updating in the Amygdala

**DOI:** 10.3389/fnmol.2021.778170

**Published:** 2021-12-02

**Authors:** Jinfeng Su, Pingping Li, Qishuai Zhuang, Xing Chen, Xiaoning Zhang, Xiaobing Li, Jingxian Wang, Xiaohan Yu, Yue Wang

**Affiliations:** ^1^Department of Neurology, Shandong Provincial Hospital Affiliated to Shandong First Medical University, Jinan, China; ^2^School of Basic Medicine, Shandong First Medical University and Shandong Academy of Medical Sciences, Jinan, China; ^3^Department of Vip Center, School and Hospital of Stomatology, Cheeloo College of Medicine, Shandong University and Shandong Key Laboratory of Oral Tissue Regeneration and Shandong Engineering Laboratory for Dental Materials and Oral Tissue Regeneration, Jinan, China

**Keywords:** fear memory, amygdala, extinction, retrieval, circRNAs, competing endogenous RNA

## Abstract

Abnormality of fear memory is one of the important pathogenic factors leading to post-traumatic stress disorder (PTSD), anxiety disorder, and other mental disorders. Clinically, although exposure therapy, which is based on the principle of fear memory extinction, has a certain effect on these diseases, it still relapses frequently in some cases. These troubles can be effectively solved by retrieving the memory in a certain time window before the extinction of fear memory. Therefore, it is generally believed that the extinction of fear memory is the result of forming new safe memory to competitively inhibit the original fear memory, while the retrieval-extinction operation is the updating or erasure of the original fear memory, thus, which has greater clinical therapeutic potential. However, what are the detailed molecular networks, specifically the circular RNAs (circRNAs), involved in fear memory updating, and the differences with fear extinction, are still unknown. In this study, we systematically observed the expression of mRNAs, microRNAs (miRNA), long non-coding RNAs (lncRNAs), and circRNAs in the basolateral amygdala of mice after fear memory formation, extinction, and updating by whole-transcriptional sequencing, then a variety of inter-group comparison and bioinformatics analysis were used to find the differential expressed RNAs, enrich the function of them, and construct the molecular interaction networks. Moreover, competing endogenous RNA (ceRNA) molecular networks and transcriptional regulatory networks for the candidate circRNAs were constructed. Through these analyses, we found that about 10% of molecules were both involved in the fear memory extinction and formation, but the molecules and their signaling pathways were almost completely different between fear memory extinction and updating. This study describes a relatively detailed molecular network for fear memory updating, which might provide some novel directions for further mechanism research, and help to develop a specific physical method for fear memory intervention, based on the regulation of these key molecules.

## Introduction

Post-traumatic stress disorder (PTSD), an increasingly common mental disease in modern society, is closely related to the inability to extinguish traumatic fear memory (Shalev et al., 2017; Series, 2019). Among the effective treatments for PTSD in the clinic, exposure therapy, which is based on the principle of fear memory extinction, is usually used in patients who are confronted with the original traumatic stimulus in a safe environment such that the emotional reaction to some “terrible” cues can be alleviated (Ostacher and Cifu, 2019). Although this behavioral treatment strategy has a certain curative effect, relatively frequent relapses of PTSD still occur. Extinction training of conditioned fear memories in the laboratory is similar to exposure therapy. After the passing of time (spontaneous recovery, SR), experiencing the aversive stimulus again (reinstatement, RI), or being in the conditioning context (renewal), fear memory is more likely to appear (Bouton, 2002; Quirk, 2002; Monfils et al., 2009). Therefore, the original fear memory is not erased but competes with a new safe memory. Since both fear memory extinction and formation depend on the construction of new memories, the molecular mechanisms involved in them are thought to be very similar.

In 2009, Monfils et al. (2009) found that if extinction training was conducted in a short period after the retrieval of fear memory, the effect of extinction might be permanent such that SR, RI, and renewal would not occur. Thus, the original fear memory has likely been erased in addition to the new learning of conditioned stimulus (CS) with a safe experience. The retrieval-extinction protocol has been shown to promote greater longer-lasting extinction in both animal and human studies, which provides insights into the treatment of some mental disorders, such as PTSD and drug addiction (Schiller et al., 2010; Xue et al., 2012; Graff et al., 2014; Jones and Monfils, 2016). Although the method was effective, the underlying mechanism remains unclear, which affects its applicability (Costanzi et al., 2011; Graff et al., 2014). Moreover, a growing number of unsuccessful repeats using the retrieval-extinction approach suggested that the contradiction will increase if the paradigms are merely imitated from the original theory without behavioral adjustment based on some objective and key indicators (Ishii et al., 2012; Luyten and Beckers, 2017; Chalkia et al., 2020). Therefore, the detailed molecular network involved in retrieval-extinction and the difference from extinction training must be delineated.

Notably, 80% of the genome is transcribed into non-coding RNAs (ncRNAs), which express in almost all parts of the body and may participate in the physiological functions and the pathological changes of these organs (The ENCODE Project Consortium, 2012). Over the past two decades, ncRNAs, which display specific spatiotemporal expression patterns across diverse species, have been extensively implicated in multiple biological processes (BPs), such as epigenetic regulation, chromatin remodeling, transcription control, and posttranscriptional processing (Eddy, 2001). A growing number of ncRNAs have been identified to play important roles in the pathogenesis of neurodegenerative disorders (Lukiw, 2013; Ma et al., 2020) and the process of fear memory formation and extinction (Lin et al., 2011; Xu et al., 2018; Malan-Muller et al., 2020). However, researchers have not determined whether and how ncRNAs are involved in retrieval-extinction-induced fear memory updating. Moreover, circular RNAs (circRNAs), as a structurally distinct class of RNA, are enriched and highly stable in the brain synapses, which predict these RNAs may serve as “memory molecules” (Zajaczkowski and Bredy, 2021), but so far fewer pieces of evidence indicate the role and regulatory mechanism of circRNAs in different stages of memory.

In this study, we collected basolateral amygdala tissues, one key area for the regulation of fear memory, from control mice and mice subjected to cued fear conditioning training, extinction training, and retrieval-extinction training. Whole transcriptome sequencing was then used to verify the differential expression profiles of ncRNAs and mRNAs during these memory processes, and specific molecular interaction pathways were subsequently screened by conducting bioinformatics analyses. Our findings indicated that some ncRNAs might be important in the processes of fear memory formation, extinction, and retrieval-extinction and clarified the associations and differences between extinction and retrieval-extinction or memory formation.

## Materials and Methods

### Animals

Specific-pathogen-free (SPF)-grade male adult (10 wk) C57BL/6J mice with a body weight of (22 ± 2) g were purchased from Vital River Laboratories (Beijing, China) and raised by special personnel in the animal room. The light/dark cycle was 12/12 h, and the mice were free to forage and drink. All operations were in accordance with the management and ethics regulations of Shandong First Medical University on the use of experimental animals. Mice were randomly divided into four groups, namely, control (C), memory formation (F) group, fear memory extinction group (E), and fear memory retrieval-extinction (RE) group.

### Behavioral Procedures

The procedures were followed previous reports with a few modifications (Clem and Huganir, 2010).

#### Cued Fear Conditioning Training

Mice were trained and tested in fear conditioning chambers (25 cm × 25 cm × 25 cm, Panlab, Harvard Apparatus, Holliston, MA, USA). Training consisted of a 3 min freely exploration of the context (Context A), followed by three repetitions of 30 s tone exposure (2 KHz, 90 dB, CS) and a foot shock at the last 1 s [0.6mA, unconditioned stimulus (US)]. The CS-US pair was presented with 60 s intervals. After the last foot shock, the mice remained in the chamber for an additional 120 s before returning to the home cage. Freezing behavior was defined as the complete absence of movement except breathing.

#### Fear Memory Test

After 24 h training, animals were exposed to a different context (context B) with fear conditioning training. After 3-min free exploration, three tones (2 KHz, 90 dB, and 30 s) were given without tone shocks. Cue presentations were separated by 60 s. The percent of freezing was calculated by freezing times/total tone exposure times × 100%.

#### Extinction Training

After 24 h training, mice were exposed to context B. After 3 min freely exploration, mice were presented with the tone (2 KHz, 90 dB, and 30 s) 18 times with 60 s intertrial interval and no foot shocks. Thirty minutes later, the same extinction training was repeated. The percent of freezing in each trial was calculated by freezing times/total tone exposure times × 100%.

#### Retrieval-Extinction Training

After 24 h training, mice received a retrieval trial in context B, in which mice were explored with a tone (2 KHz, 90 dB) for 30 s without foot shocks. Then the mice were returned to their home cage for 1 h, after which they received the extinction training.

#### Spontaneous Recovery Test

About 10 days after retrieval-extinction training, mice were tested for fear memory by exposing them to the context B for three tones (2 KHz, 90 dB, and 30 s) treatment. The percent of freezing was calculated by freezing times/total tone exposure times × 100%.

#### Reinstatement Test

One day after retrieval-extinction training, mice received two unsignaled footshocks (0.6 mA) in context B without tone presentation. The next day, they were tested for fear memory by exposure to the tone (2 KHz, 90 dB, and 30 s) three times without foot shocks in context B. The percent of freezing was calculated by freezing times/total tone exposure times × 100%.

#### Renewal Test

For the renewal experiment, mice acquired fear in context A, then retrieved and extinguished in context B. About 24 h later, mice were tested for fear renewal in context A for three tones (2 KHz, 90 dB, and 30 s) treatment. The percent of freezing was calculated by freezing times/total tone exposure times × 100%.

### Tissue Collection and RNA Isolation

One hour after fear conditioning training (F group) or the last extinction training (E and RE group), mice were anesthetized with isoflurane, then their brains were quickly collected and placed in a mouse brain slicer (RWD, China). Coronal sections (1-mm thick) from −0.58 to −2.06 mm (anterior-posterior coordinate relative to bregma) were collected and most of the basolateral part of the amygdala was isolated following delineations from the mouse brain atlas under a dissecting microscope (refer to Supplementary Figure S1). The tissue was dissected on ice and stored at −80°C until use. The control group performed the same procedure as the F group except that they were not given foot shocks. The tissues of control mice were collected at the same time as the F group.

Total RNA was extracted from the frozen tissues with TRIzol reagent (Life Technologies, Carlsbad, CA, USA). The RNA concentration of each sample was measured by NanoDrop ND-1000 (Thermo, Waltham, MA, USA). The RNA samples will pass the quality control if a qualifying ratio of OD260 to OD280 is in the range of 1.8–2.1. The RNA sample integrity and gDNA contamination were determined by denaturing agarose gel electrophoresis.

### RNA Library Preparation and Sequencing

RNA library preparation and high throughput sequencing were performed by Cloud-Seq Biotech (Shanghai, China). Briefly, for mRNAs, long non-coding RNAs (lncRNAs), and circRNAs sequencing, total RNA was firstly removed from the rRNAs with NEBNext rRNA Depletion Kit (New England Biolabs, Inc., Ipswich, MA, USA) according to the instructions of the manufacturer. Then, rRNA-depleted RNA was used to construct the RNA libraries by using NEBNext® Ultra™ II Directional RNA Library Prep Kit (New England Biolabs, Inc., Ipswich, MA, USA) following the instructions of the manufacturer. After that, BioAnalyzer 2100 system (Agilent Technologies, Inc., Santa Clara, CA, USA) was used to evaluate the quality and quantity of Libraries. Library sequencing was performed on Hiseq 4000 (Illumina, San Diego, CA, USA) with 150 bp paired-end reads.

For microRNA (miRNA) sequencing, the total RNA of each sample was used to prepare the miRNA sequencing library following next steps: (1) 3′-adaptor ligation; (2) 5′-adaptor ligation; (3) cDNA synthesis; (4) PCR amplification; and (5) size selection of ~150 bp PCR amplicons. The libraries were denatured as single-stranded DNA molecules, captured on Illumina flow cells, amplified *in situ* as clusters, and finally sequenced for 50 cycles on Hiseq 4000 (Illumina, San Diego, CA, USA). All data had been uploaded to GEO (GSE 185808, https://www.ncbi.nlm.nih.gov/geo/query/acc.cgi?acc=GSE185808).

### Data Processing

After sequencing on the Illumina HiSeq 4000 sequencer, paired-end reads were harvested. Q30 was used for quality control, and cutadapt1 software was applied to remove joints, remove low-quality reads, and obtain high-quality reads. For circRNA analysis, STAR software (v2.5.1.b, Cold Spring Harbor Laboratory, New York, NY, USA) was used to compare high-quality reads to the reference genome/transcriptome, and DCC software (v0.4.4, Dieterich Lab, Heidelberg, Germany) was applied for circRNA detection and identification. CircBase database and Circ2Traits were applied to annotate the identified circRNAs. For lncRNA and mRNA analysis, HISAT2 software (v2.0.4, Kim Lab, Texas, TX, USA) was applied to compare high-quality reads to the mouse reference genome (UCSC mm10). Then, under the guidance of the gtf gene annotation file, the software cuffdiff (v2.2.1, Cole Trapnell's Lab, Washington, DC, USA) was applied to obtain the fragments per kilobase of exon per million fragments mapped (FPKM) values of lncRNA and gene-level mRNAs at the transcript level, which were used as the expression profiles of lncRNA and mRNAs, and the multiple changes and *p*-values between the two samples were calculated to screen the differentially expressed lncRNAs (DELs) and mRNAs. The steps of miRNA analysis were as follows. After sequencing with illumine sequencer, image analysis, and base recognition, the original reads after quality control were harvested. Then, Q30 was used for quality control, and Cutadapt software (v1.9.3, NBIS, Uppsala, Sweden) was applied to delink the original reads, remove low-quality reads, and retain reads with a length ≥15 nt to obtain the reads after delinking (i.e., trimmed reads). Then, the timed reads of all samples were merged, and the miRDeep2 software (v2.0.0.5, MDC, Berlin, Germany) was applied to predict the new miRNA. Novoalign software (v3.02.12, Novocraft Technologies Sdn Bhd, Selangor, Malaysia) was used to align the trimmed reads of each sample to the combined mouse pre-miRNAs database. The number of tags compared to each mature miRNA was counted as the original expression level of the miRNA, and the tag counts per million aligned miRNAs (TPM) method was used for standardization.

### Gene Ontology and Kyoto Encyclopedia of Genes and Genomes Pathway Enrichment Analyses

The lists of differentially expressed genes (DEGs), DELs, differentially expressed circRNAs (DECs), and differentially expressed miRNAs (DEMs) between different groups of mice were generated using the edgeR software, where statistical significance was set as log_2_ |FC| ≥ 1 and *p* < 0.05. Then, the DAVID database (http://david.ncifcrf.gov/), which is a commonly available database for gene enrichment and functional annotation analyses, was used. This database integrates biological data and analytical tools to afford systematic and comprehensive annotation of biological functions for large-scale lists of genes or proteins. The GO annotation and KEGG pathway enrichment analyses of the identified genes were performed using DAVID. A visual network analysis of the KEGG analysis results was conducted using Cytoscape software (v3.6.1).

### Weighted Gene Co-expression Network Analysis

Weighted gene co-expression network analysis is a systematic biological method used to establish a scale-free network according to gene expression profiles. To establish the system, a similarity matrix that calculated the absolute value of Pearson's correlation coefficient between two genes was established using expression data. Then, the similarity matrix was converted into an adjacency matrix, where the β value was the soft-threshold to strengthen strong connections and ignore weak correlations between genes in the adjacency matrix. The Dynamic TreeCut algorithm was used to distinguish network modules. The most representative genes were the module eigengenes (MEs), which represented the overall level of gene expression in individual modules. Module membership (MM) was analyzed using Pearson's correlation coefficient of the expression profile of one gene in all samples and one ME. Finally, the gene significance (GS) was applied to estimate the gene with other biological properties, the higher the value of GS, the greater the correlation between the gene and the properties. The MM was used to analyze the correlation between the genes with the module, the higher the value of MM, the greater the correlation between the gene and the module. Therefore, the expression profile of differentially expressed mRNAs was applied to establish a free-scale network and identify significant modules related to properties to analyze the differential genes in these modules.

### Establishment of the Protein-Protein Interaction and circRNA-miRNA-mRNA Network

Search tool for the Retrieval of Interacting Genes/Proteins is a search tool that can analyze the interaction relationship between proteins. The PPI interaction networks between the DEGs were constructed by Search Tool for the Retrieval of Interacting Genes (STRING) database. Cytoscape software (v3.6.1) was applied to screen hub genes based on the degrees.

The DEG-associated upstream regulator miRNAs were predicted by two miRNA databases (miRDB, Targetscan). The selected miRNAs were then overlapped with the DEMs, and the negative interaction pairs between DEMs and DEGs were applied to construct the miRNA-mRNA network using Cytoscape software. The DEM-related circRNAs, predicted by the TargetScan website, were integrated with the DECs. Then, the identified circRNAs were integrated with the miRNA-mRNA interactions to construct the DEG-DEM-DEC competing endogenous RNAs (ceRNAs) network using Cytoscape software. The transcription factor (TF) of the related circRNAs was predicted by the CircBase website.

### Prediction of TFs Binding to Hub circRNAs

The potential TFs binding to hub circRNAs in ceRNA network were predicted through the JASPAR website (http://jaspar.genereg.net/). Depending on the number of binding sites between TFs and hub circRNAs, the top eight TFs were shown, and the key TFs were incorporated into the ceRNA networks, thus the TF-ceRNA networks were formed.

### Statistical Analysis

Two normally distributed groups were compared by using a *t*-test. Parameters for the high-throughput sequencing-related data were calculated, and statistical computing was performed by using R software. All data were expressed as mean ± SD, a *p* < 0.05 was considered statistically significant.

## Results

### Identification and Functional Annotations of the RNAs That are Possibly Involved in Memory Formation and Extinction

**Figure 1:**
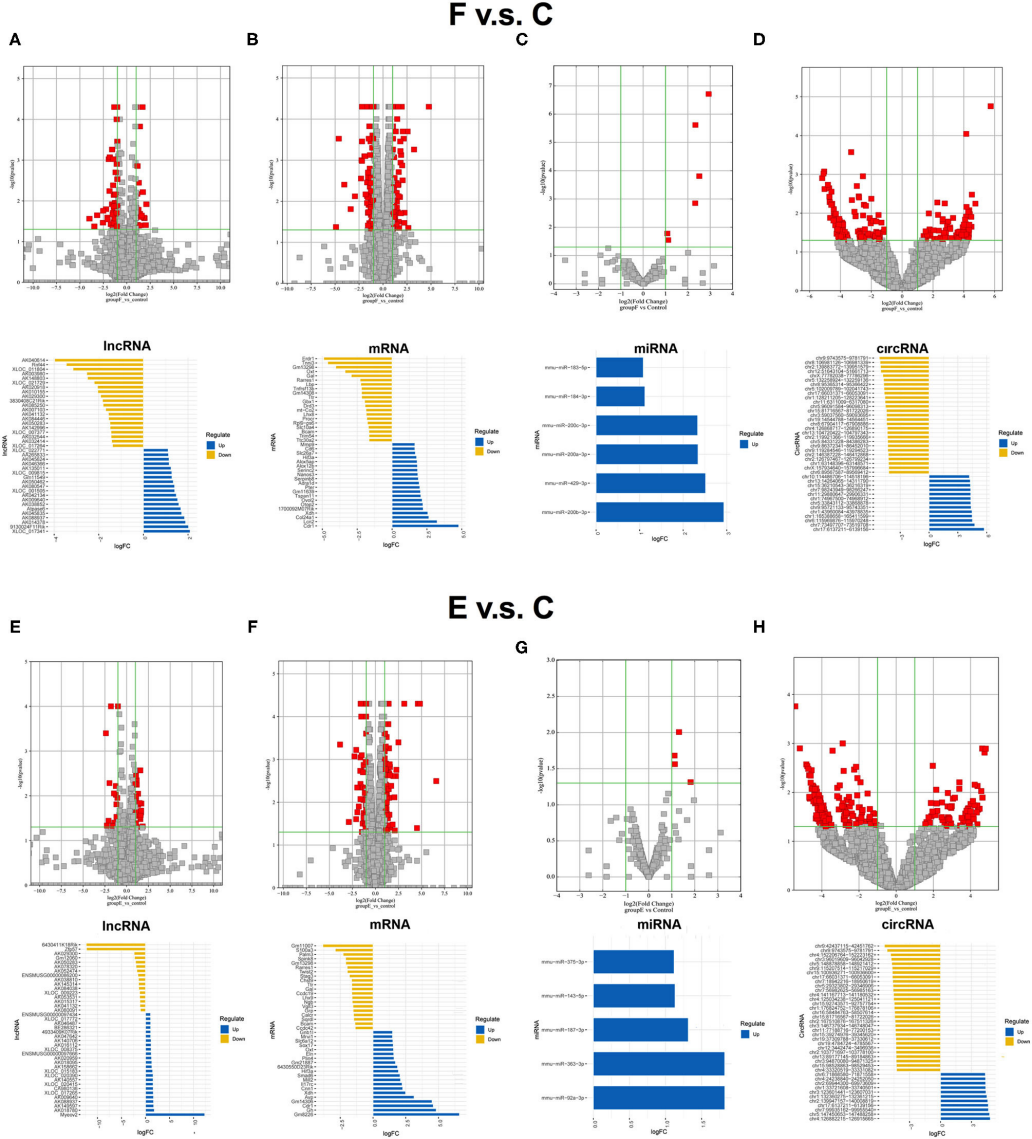
Expression profiles of distinct RNAs between F and control groups, and E and control groups. **(A)** In the volcano plots, red and gray points represented abnormal lncRNAs and non-significantly DELs, respectively, in F and control groups. The top 20 upregulated genes (blue) and the top 20 downregulated (yellow) genes were presented. Expression profiles were similarly shown for mRNA **(B)**, miRNA **(C)**, and circRNA **(D)**. **(E)** In the volcano plots, red and gray points represented abnormal lncRNAs and non-significantly differentially expressed lncRNAs, respectively, in **(E)** and control groups. The top 20 upregulated genes (blue) and the top 20 downregulated (yellow) genes were presented. Expression profiles were similarly shown for mRNA **(F)**, miRNA **(G)**, and circRNA **(H)**. F, fear memory formation group. E, fear memory extinction group. miRNA, micro-RNA; circRNA, circular RNA; lncRNAs, long non-coding RNAs.

Before the whole transcriptome sequencing was performed, we first confirmed the effects of our different training paradigms on fear memory by analyzing the duration of mice freezing (refer to Supplementary Figure S2). After proving the feasibility of these training paradigms, additional three mice in each group were trained, respectively, once more, and BLA samples were collected at a specific time point for whole transcriptome sequencing. The differentially expressed RNAs between the F group and the control group were screened to identify the RNAs that are possibly involved in memory formation. Seventy-one significantly dysregulated lncRNA transcripts (such as 21 upregulated transcripts and 50 downregulated transcripts), 151 dysregulated mRNA transcripts (70 upregulated and 81 downregulated), 6 upregulated miRNA transcripts, and 232 dysregulated circRNA transcripts (76 upregulated and 156 downregulated) were identified between the F and control groups, and top 20 candidates were showed respectively (Figures 1A–D). Then, the differentially expressed RNAs between the E group and the control group were analyzed. Forty-five significantly dysregulated lncRNA transcripts (such as 27 upregulated transcripts and 18 downregulated transcripts), 130 dysregulated mRNA transcripts (76 upregulated and 54 downregulated), 5 upregulated miRNA transcripts, and 243 dysregulated circRNA transcripts (70 upregulated and 173 downregulated) were differentially expressed between the E group and the control group (Figures 1E–H). By further analyzing the similarities and differences of dysregulated RNAs between the control group and fear memory formation group or extinction group, we found that the common changed RNAs accounted for 10.78% of the total changed RNAs in these two processes (Supplementary Figures S3A–C), such as 7 significantly dysregulated lncRNAs (2 upregulated, 4 downregulated, and 1 reverse), 33 dysregulated mRNAs (10 upregulated, 19 downregulated, and 4 reverse), 0 dysregulated miRNAs, and 46 dysregulated circRNAs (19 upregulated and s27 downregulated), suggesting that the processes of fear memory formation and extinction might share some similar molecular mechanisms, but the proportion is relatively low.

**Figure 2:**
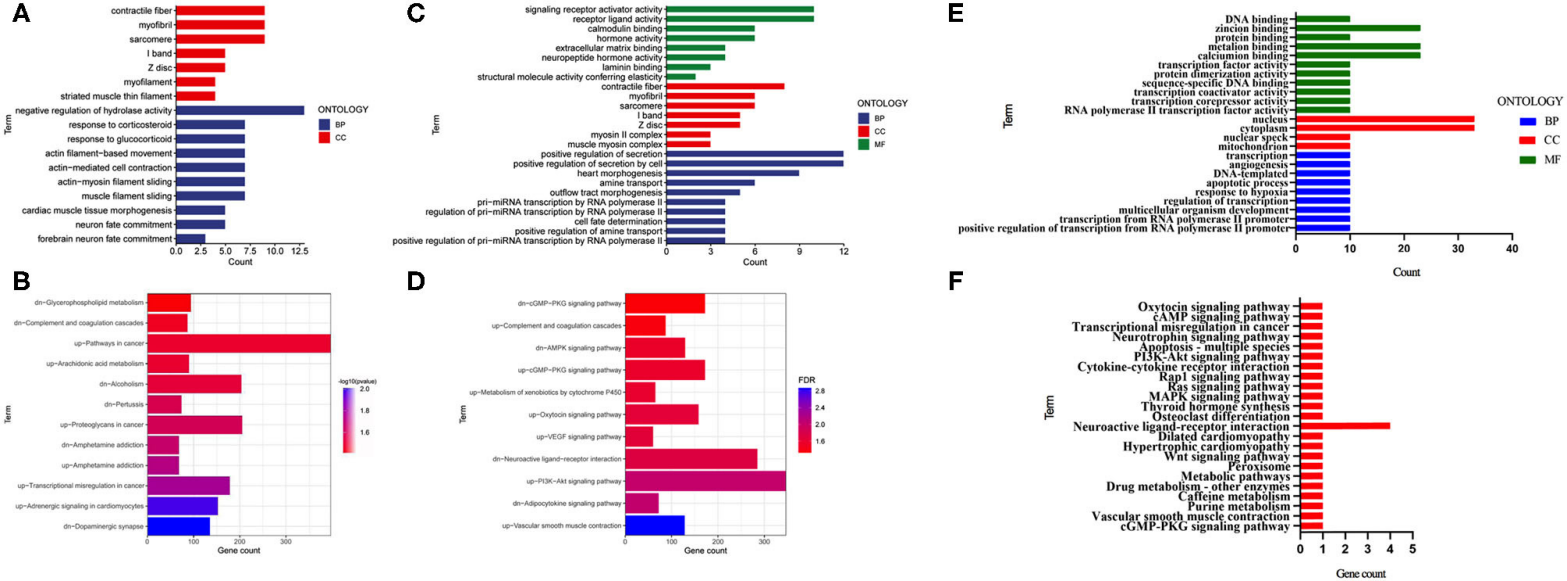
GO and KEGG enrichment analyses were performed for the mRNAs changed after fear memory formation and extinction. **(A,B)** The DEGs in F and C groups were analyzed by GO and KEGG. **(C,D)** The DEGs in E and C groups were analyzed by GO and KEGG. **(E,F)** The commonly changed mRNAs after fear memory formation and extinction were analyzed by GO and KEGG. BP, biological process; CC, cellular component; MF, molecular function; GO, gene oncology; KEGG, Kyoto Encyclopedia of Genes and Genomes; DEGs, differentially expressed genes.

To clarify the biological functions and signaling pathways of these DEGs, we further performed GO and KEGG enrichment annotations of the DEGs in the E, F with control groups. Fifty significant GO-BPs and 7 significant GO-cellular components (CCs) were obtained from the GO functional annotation analysis of 151 differentially expressed mRNAs between the F and control groups. The top 10 GO-BP and GO-CC terms are presented in Figure 2A. Regarding the KEGG pathway enrichment analysis, 12 significant KEGG pathways were enriched in these 151 mRNAs, among which upregulated genes were enriched in six pathways and downregulated genes were enriched in six pathways (Figure 2B). Subsequently, 131 DEGs identified between the E and control groups were subjected to GO functional annotation. Seventy-eight significant GO-BPs, seven significant GO-CCs, and eight significant GO-molecular functions (MF) were obtained using *p* < 0.05 as the screening condition. The top 10 GO-BP, GO-CC, and GO-MF terms are presented in Figure 2C. In the KEGG pathway analysis, 11 significant KEGG pathways were enriched, among which upregulated genes were enriched in 7 pathways, and downregulated genes were enriched in 4 pathways (Figure 2D). Next, the mRNAs that changed after both fear conditioning and fear extinction were subjected to GO and KEGG enrichment annotations. Ten significant GO-BPs, four significant GO-CCs, nine significant GO-MFs, and 12 significant KEGG pathways were annotated (Figures 2E,F). The regulation of transcription and neuroactive ligand-receptor interaction were both significantly enriched in the processes of fear formation and extinction, suggesting that fear memory extinction was also assisted with neural cell activation and new protein synthesis, similar to memory formation.

### The Differences in RNA Expression and Pathway Activation Between the E and RE Groups

**Figure 3:**
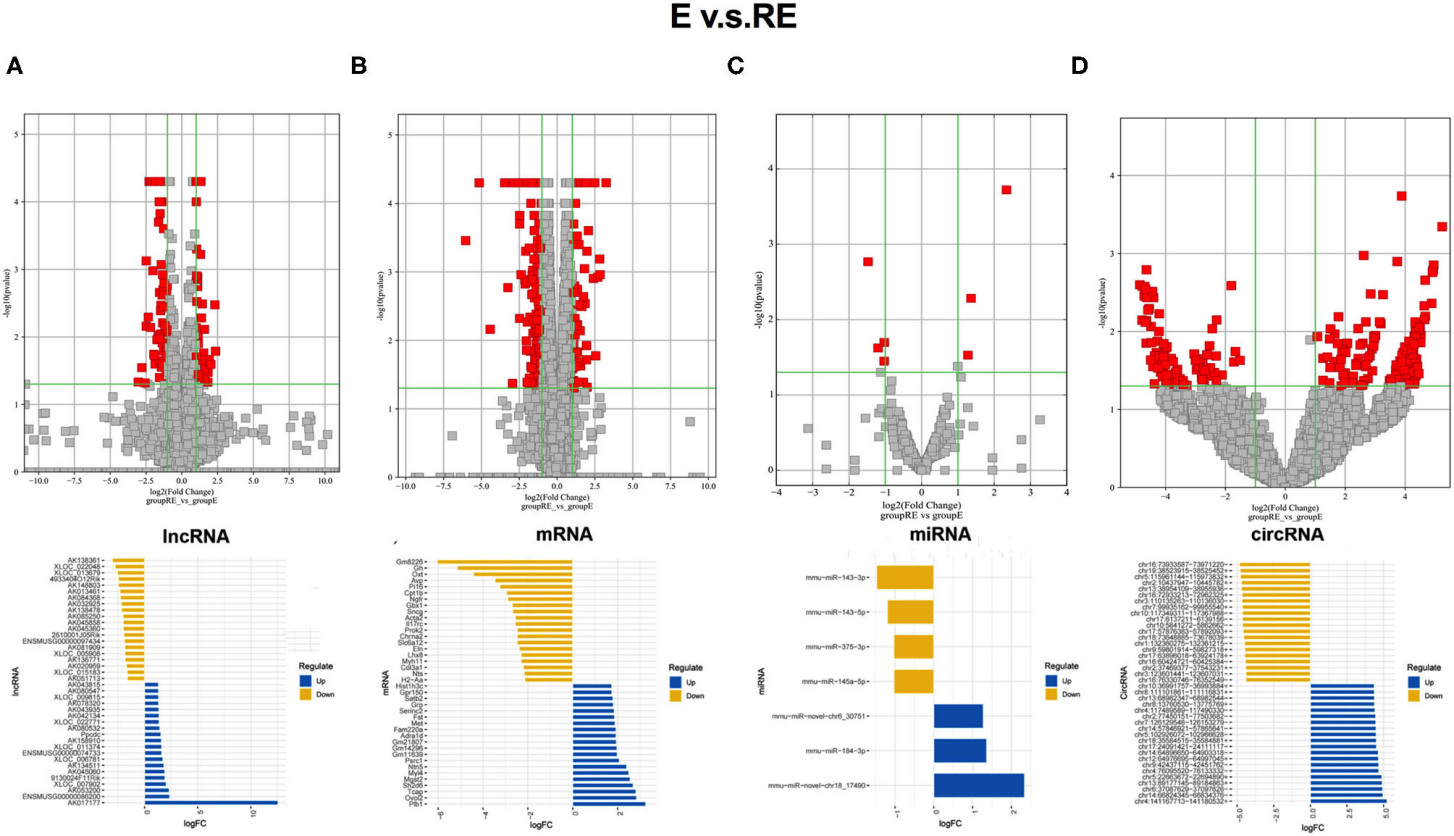
Different changed RNAs between E and RE groups. **(A)** In the volcano plots, red and gray points represented abnormal lncRNAs and non-significantly differentially expressed lncRNAs, respectively, when E compared with the RE group. The top 20 upregulated genes (blue) and the top 20 downregulated (yellow) genes were presented. Expression profiles were similarly shown for mRNA **(B)**, miRNA **(C)**, and circRNA **(D)**. E, fear memory extinction group. RE, retrieval-extinction group. circRNA, circular RNA; lncRNAs, long non-coding RNAs.

The differentially expressed RNAs between the E group and RE group were analyzed to understand the differences in molecular mechanisms between the fear memory extinction and updating groups. One hundred and eight dysregulated lncRNA transcripts (43 upregulated and 65 downregulated), 295 dysregulated mRNA transcripts (79 upregulated and 216 downregulated), 7 dysregulated miRNA transcripts (3 upregulated and 4 downregulated), and 256 dysregulated circRNA transcripts (159 upregulated and 97 downregulated) were identified between the E group and RE group (Figures 3A–D).

**Figure 4:**
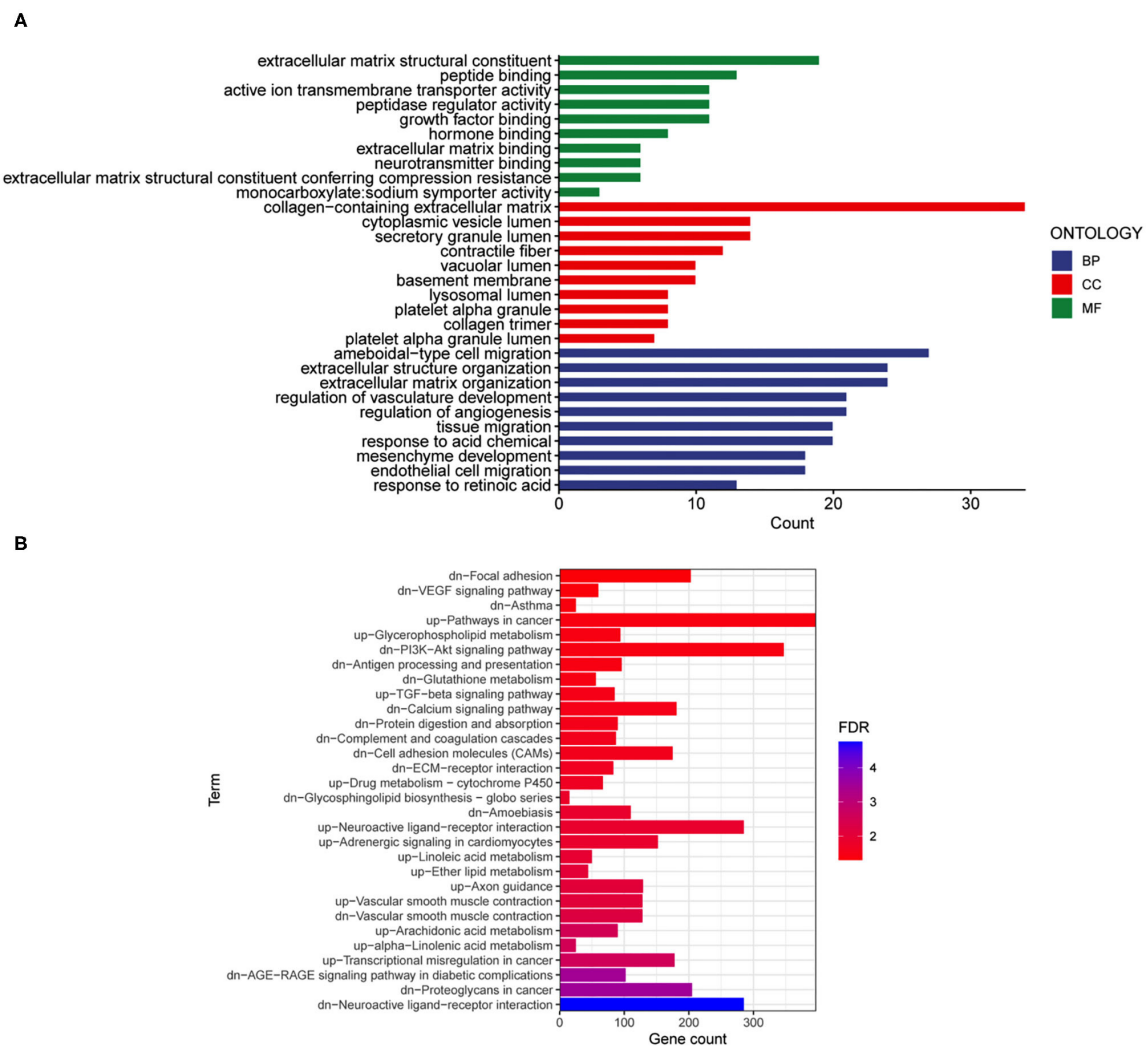
Gene ontology and KEGG enrichment analyses were performed for the mRNAs changed after fear memory extinction and updating. **(A,B)** The DEGs in E and RE groups were analyzed by GO **(A)** and KEGG **(B)**. GO, gene oncology; KEGG, Kyoto Encyclopedia of Genes and Genomes; DEGs, differentially expressed genes.

Then, the 295 DEGs identified between the E and RE groups were applied for GO functional annotation. A total of 336 significant GO-BP, 17 significant GO-CC, and 43 significant GO-MF terms were obtained using *p* < 0.05 as the screening condition. The top 10 GO-BP, GO-CC, and GO-MF terms are presented in Figure 4A. Moreover, 30 significant KEGG pathways were enriched in the 295 genes, among which upregulated genes were enriched in 13 pathways and downregulated genes were enriched in 17 pathways (Figure 4B). The PI3K-AKT signaling pathway, neuroactive ligand-receptor interaction, and calcium signaling pathway were the top three neural function-related signaling pathways, suggesting that they are deeply involved in fear memory updating. Finally, the common elements in “RE vs. control” and “E vs. control” comparisons were analyzed. The results showed that only genes related to neuroactive ligand-receptor interactions were enriched (Table 1).

**Table 1:** Common element in “RE vs. C” and “E vs. C.”

**RE vs. C**				**E vs. C**			
	**Term**	***P*-value**	**Genes**		**Term**	***P*-value**	**Genes**
Down	Neuroactive ligand-receptor interaction	3.24752E-08	CALCR//CHRM5// CHRNA2//CRHR2// GABRQ//GALR1//GLP1R// GLRA1//NPY2R//OXTR// PRLR//PTH2R//RXFP3// TRHR	Down	Neuroactive ligand-receptor interaction	0.01892288	CALCR//HCRTR1//TRHR

### Gene Co-expression Network Construction and Module Identification

**Figure 5:**
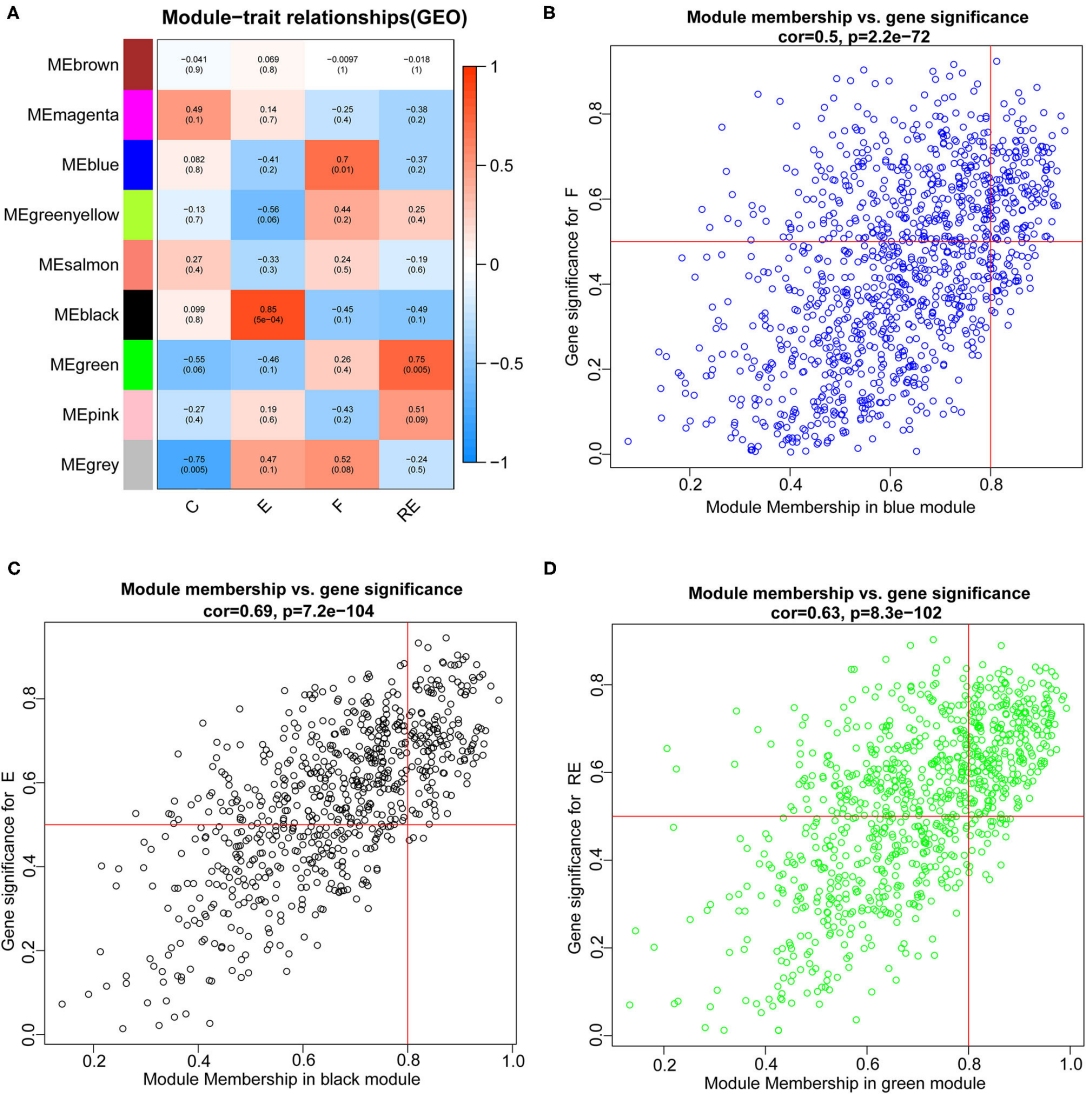
Identification of modules associated with the F, RE, and E in mice. **(A)** Heatmap of the correlation between the module and F, RE, and E in mice. We selected blue, black, and green modules for subsequent analysis. Scatter plot analysis of modules in the blue **(B)**, black **(C)**, and green **(D)** modules.

Weighted gene co-expression network analysis was carried out to identify co-expression networks of genes associated with different stages of fear memory. The phylogenetic tree was constructed using the gene expression matrix of 12 mice. The sample dendrogram and trait heatmap are presented in Supplementary Figure S4A. The scale independence and mean connectivity analysis showed that when the weighted value equaled 14, the average degree of connectivity was close to 0, and scale independence was >0.8; therefore, the weighted value was set to 14 (Supplementary Figure S4B). By calculating the correlation coefficients between genes, the genes were theoretically classified based on the expression pattern, and the patterned genes were divided into one module. Nine co-expressed modules were identified (Supplementary Figure S4C). Pearson's correlation coefficient between the module and each phenotype was calculated to verify the modules most significantly associated with different stages of fear memory (Figure 5A). The strongest association in the module-trait relationship was detected between the black module and E group (*r* = 0.85, *p* = 5e−04). The second strongest association in the module-phenotype relationship was observed between the green module and RE group (*r* = 0.75, *p* = 0.005). The third strongest association in the module-phenotype relationship was observed between the blue model and F group (*r* = 0.7, *p* = 0.01). The labeledHeatmap function was applied to calculate the correlation coefficients for MM with significant genes in the blue, black, and green modules to identify the correlation between the module of interest and memory features. The results revealed a significant correlation of MM with significant genes in the blue, black, and green modules in the F (cor = 0.5, *p* = 2.2e−72), E (cor = 0.69, *p* = 7.2e−104), and RE (cor = 0.63, *p* = 8.3e−102) groups (Figures 5B–D). Thus, blue, black, and green modules were selected as modules of interest for subsequent analyses.

### Analysis of Important DEGs in the E and RE Groups

**Figure 6:**
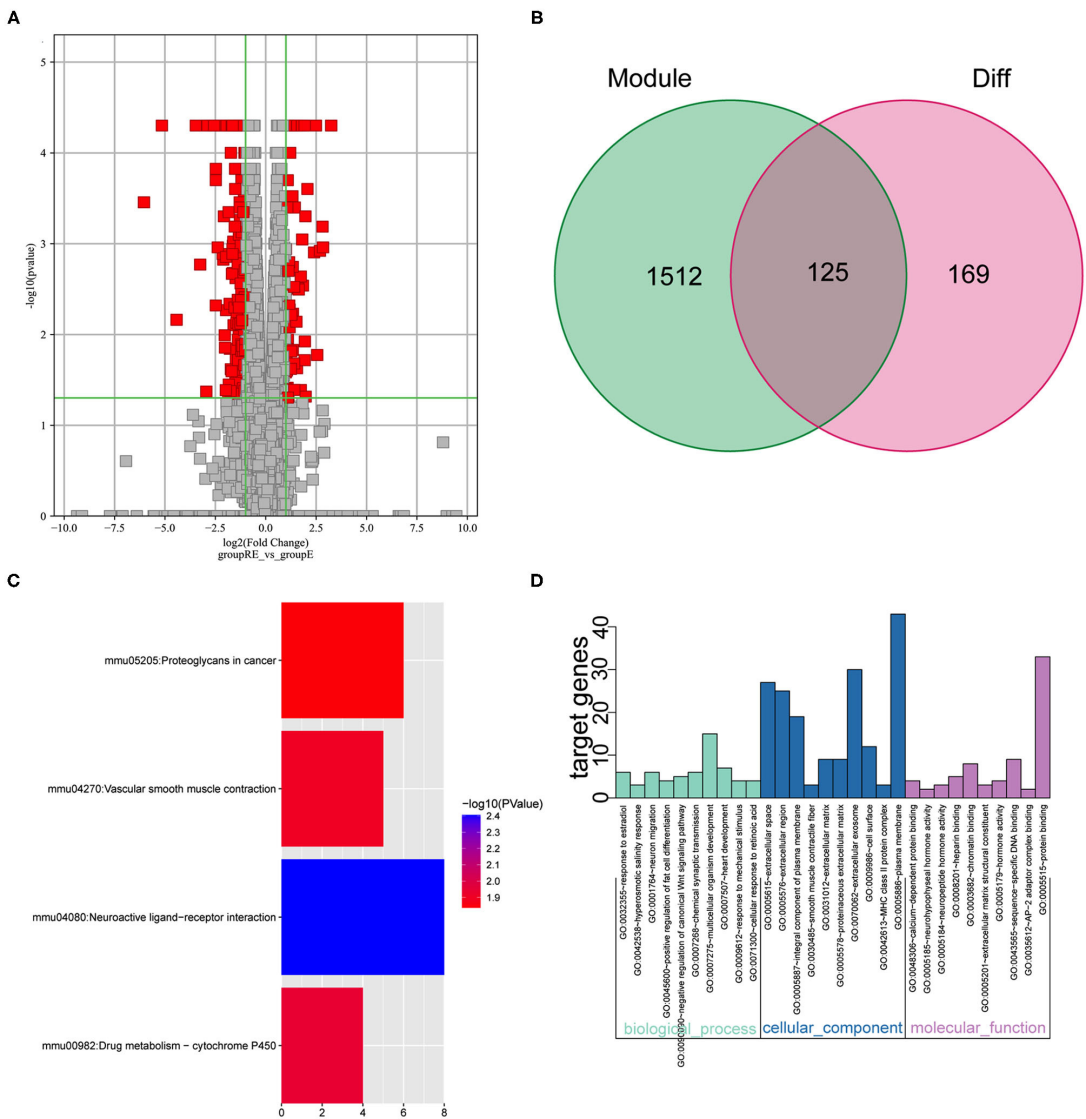
Analysis of key genes in E and RE groups. **(A)** The DEGs in E and RE groups were analyzed. **(B)** The obtained mRNAs were presented by the Venn diagram. **(C)** Four significant pathways were obtained by KEGG enrichment analysis of 125 genes. **(D)** The top 10 GO-BP, GO-CC, and GO-MF were obtained by GO annotation analysis of 125 genes. E, fear memory extinction group. RE, retrieval-extinction group; DEGs, differentially expressed genes; GO-BP, gene oncology-biological process; GO-CC, gene oncology-cellular component; GO-MF, gene oncology-molecular function; KEGG, Kyoto Encyclopedia of Genes and Genomes.

We performed the analysis described below to further understand the differences involved in memory extinction and retrieval-extinction. First, genes in the black and green modules described above were merged, and 1,637 genes were obtained. Then, these 1,637 genes were integrated with 296 DEGs from the RE and E groups, and 125 DEGs were obtained (Figures 6A,B). Subsequently, 125 DEGs were subjected to KEGG pathway enrichment analysis and GO functional annotation. Four meaningful pathways, 57 significant GO-BP, 16 significant GO-CC, and 10 significant GO-MF terms were obtained, and the top 10 elements are displayed (Figures 6C,D). Subsequently, 125 DEGs were imported into the STRING database to obtain their corresponding protein interaction information. DEGs with scores ≥0.4 were selected to construct a visual network model with 125 nodes and 163 edges (Supplementary Figure S5A). The protein interaction files were imported into Cytoscape to construct the protein interaction network of the target. Topologically important nodes in the interaction network were chosen using the Maximal Clique Centrality (MCC) algorithm in the CytoHubba plug-in, and the top 10 genes (*Avp, Htr2c, Oxt, Nts, Adra1d, Tacr3, P2ry1, Fn1, Col1a1*, and *Dcn*) in terms of the MCC score were selected as the core genes of the network to further select the important DEGs between the E and RE groups (Supplementary Figure S5B). The expression levels of the 10 hub DEGs in the E and RE groups are presented in Supplementary Figure S5C. The expression of Adra1d was upregulated in the RE group compared with that in the E group, and the expression of the other genes was downregulated in the RE group. The ClueGo and Cluepedia plug-ins in Cytoscape version 3.9.0 were used for GO functional annotation and KEGG pathway enrichment of hub DEGs. Using *p* ≤ 0.05 as the screening condition, 17 significant GO-BP terms were obtained (Table 2), but no significant GO-CC terms, GO-MF terms, or KEGG pathways were enriched.

**Table 2:** GO annotation on the 10 hub genes in E and RE groups.

**GOTerm**	***P*-value**	**Associated genes**
Excretion	2.50E-06	Adra1d, Avp, Oxt
Hyperosmotic response	2.15E-07	Avp, Oxt, Tacr3
Mating	2.90E-06	Avp, Oxt, P2ry1
Response to salt stress	5.25E-07	Avp, Oxt, Tacr3
Positive regulation of blood circulation	7.58E-08	Adra1d, Avp, Htr2c, Tacr3
Copulation	2.99E-07	Avp, Oxt, P2ry1
Positive regulation of organic acid transport	1.52E-06	Avp, Htr2c, Oxt
Hyperosmotic salinity response	4.14E-08	Avp, Oxt, Tacr3
Penile erection	7.15E-08	Avp, Oxt, P2ry1
Positive regulation of blood pressure	1.09E-08	Adra1d, Avp, Oxt, Tacr3
Positive regulation of muscle contraction	1.92E-06	Adra1d, Oxt, Tacr3
Positive regulation of amine transport	1.81E-06	Avp, Htr2c, Oxt
Positive regulation of anion transport	3.82E-06	Avp, Htr2c, Oxt
Positive regulation of smooth muscle contraction	5.71E-07	Adra1d, Oxt, Tacr3
Neuropeptide hormone activity	2.69E-07	Avp, Nts, Oxt
Regulation of smooth muscle contraction	3.65E-06	Adra1d, Oxt, Tacr3
Positive regulation of vasoconstriction	1.19E-06	Adra1d, Avp, Htr2c

### Identification of the circRNA-Associated ceRNA Network Related to the DEGs in the E and RE Groups

The miRDB and TargetScan databases were used to predict the interactions between the above DEGs and miRNAs in the E and RE groups. A total of 439 miRNAs of 10 hub DEGs were predicted by the two websites. Then, eight DEMs were obtained under the screening conditions of log_2_|FC| ≥ 0.58 and *p* < 0.05, of which four were upregulated and four were downregulated in the RE group (Supplementary Figure S6A). Two common miRNAs, mmu-miR-200a-3p and mmu-miR-143-3p, were obtained by intersecting the predicted miRNAs with the DEMs in the E and RE groups. Among them, mmu-miR-200a-3p was upregulated in the RE group, and mmu-miR-143–3p was downregulated (Supplementary Figure S6B). Next, a differential circRNA analysis was performed on the RE and E groups, and 256 circRNAs were significantly differentially expressed under the screening conditions of log_2_|FC| ≥ 1 and *p* < 0.05, among which 159 circRNAs were upregulated and 97 circRNAs were downregulated in the RE group. The TargetScan website was used to predict the regulatory circRNA of mmu-miR-200a-3p and mmu-miR-143-3p, and 14 circRNAs were obtained. After intersecting the predicted circRNAs with the DECs, 2 downregulated circRNAs corresponding to mmu-miR-200a-3p and 12 upregulated circRNAs corresponding to mmu-miR-143-3p were obtained (Supplementary Figure S6C). The ceRNA network composed of 14 circRNAs, 2 miRNAs, and 4 mRNAs that were differentially expressed in the E and RE groups is presented in Supplementary Figure S6D. These findings revealed that ncRNAs might also play an important role in memory updating through ceRNA mechanisms.

Subsequently, we predicted the possible TFs that regulate circRNA expression by JASPAR. Eight TFs (BARHL1, DLX1, GATA1, HOXA5, NKX2–5, PAX2, PRRX2, and SOX10) were identified for these 14 key circRNAs, and the number of binding sites between the TFs and these 14 circRNAs is displayed (Supplementary Figures S7A–H). Then, these eight TFs were incorporated into the regulatory ceRNA network (Supplementary Figure S8).

### Analysis of Crucial DEGs in the F and E/RE Groups

**Figure 7:**
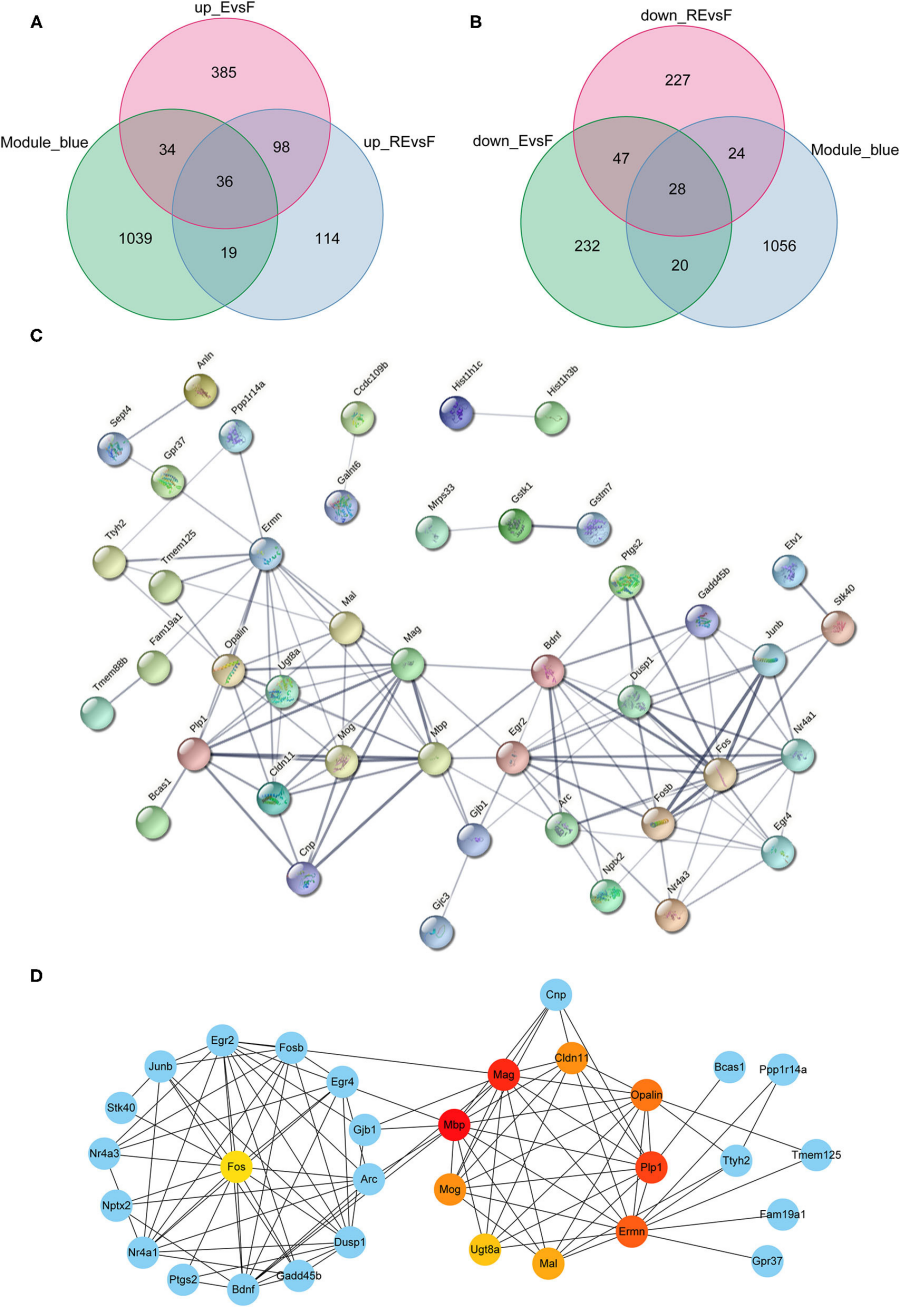
Analysis of key genes in F and E/RE groups. **(A,B)** The obtained mRNAs were presented by Venn diagram. **(C,D)** PPI network was established by using the obtained mRNAs. F, Fear memory formation group. E, Fear memory extinction group. RE, retrieval-extinction group; PPI, protein-protein interaction.

Although the weakening effect of fear memory differs between fear extinction and the RE intervention, they are opposite to memory formation. Therefore, studies exploring the differences between fear memory formation and memory extinction are very important. Since the blue module showed a positive correlation with the F group but a negative correlation with the E and RE groups, we chose the blue module to analyze the key modules between the F and E/RE groups, and this module included 1,128 mRNAs. Meanwhile, the DEGs between the F and RE groups and between the F and E groups were screened. The upregulated genes and downregulated genes obtained from the two groups intersected with the genes in the blue module, and 64 common genes were obtained, i.e., 36 upregulated genes and 28 downregulated genes (Figures 7A,B). The 64 genes were imported into the STRING website for the protein interaction analysis, and their corresponding protein interaction information was obtained. Genes with scores ≥0.4 were selected to construct a visual network model with 64 nodes and 114 edges (Figure 7C). The protein interaction files were imported into Cytoscape to construct the protein interaction network of the target. The topologically important nodes in the interaction network were screened using the MCC algorithm in the CytoHubba plug-in. The top 10 genes in terms of the MCC score were selected as the core genes of the network (*Mbp, Mag, Plp1, Ermn, Opalin, Cldn11, Mog, Mal, Ugt8a*, and *Fos*), and visualization was performed (Figure 7D). The differential expression analysis showed that the expression of *Mbp, Mag, Plp1, Ermn, Opalin, Cldn11, Mog, Mal*, and *Ugt8a* was upregulated in the RE and E groups, while the expression of *Fos* was downregulated. The ClueGo and Cluepedia plug-ins in Cytoscape version 3.9.0 were used to annotate the hub genes for GO functions and enrichment of KEGG pathways. Using *p* ≤ 0.05 as the screening condition, five meaningful GO-BP terms and three meaningful GO-CC terms were obtained, but no meaningful GO-MF terms and KEGG pathways were enriched. According to the GO functional annotation, hub genes were involved in the formation of the central nervous system and the process of protein localization in synapses (Table 3).

**Table 3:** GO annotation on the 10 hub genes in F and E/RE groups.

**Ontology**	**GOTerm**	***P*-value**	**Associated genes**
BP	Protein localization to axon	1.49E-05	Mal, Ugt8a
BP	Protein localization to paranode region of axon	4.01E-06	Mal, Ugt8a
BP	Axon ensheathment in central nervous system	5.70E-05	Mag, Plp1
BP	Oligodendrocyte development	2.31E-04	Mag, Plp1
BP	Central nervous system myelination	5.70E-05	Mag, Plp1
CC	Internode region of axon	1.15E-06	Ermn, Mbp
CC	Paranode region of axon	2.59E-05	Ermn, Mag
CC	Compact myelin	3.62E-05	Mag, Mbp

### Identification of the circRNA-Associated ceRNA Network Related to the DEGs in the F and E/RE Groups

**Figure 8:**
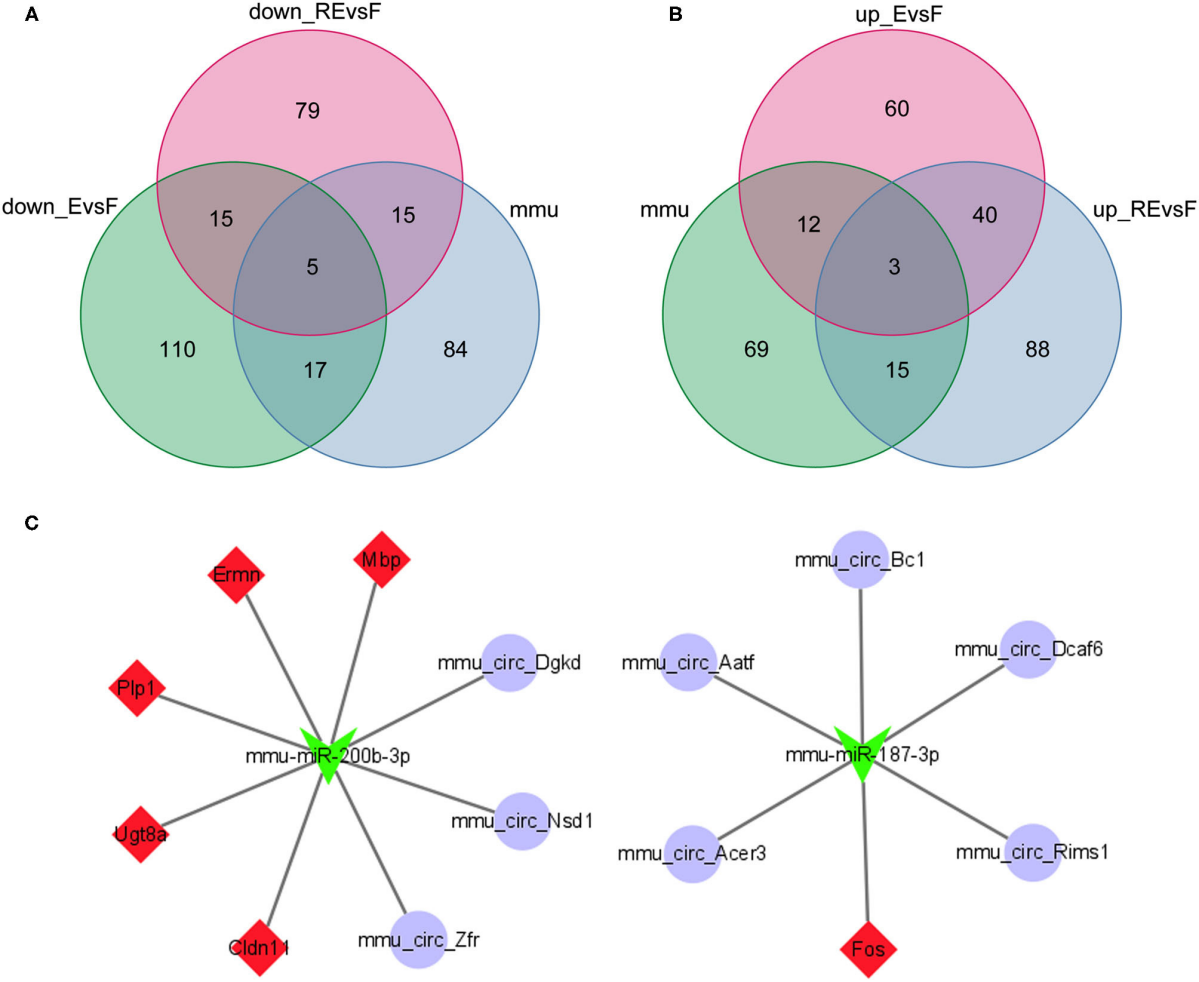
Two circRNA-associated networks were established between F and E/RE. **(A,B)** The obtained circRNAs were presented by the Venn diagram. **(C)** The circRNA-associated ceRNA networks in F and E/RE. The purple circles represented circRNAs, the green arrowhead represented miRNAs, and the red diamonds represented target mRNAs. Network edges represented competitive interactions. F, fear memory formation group. E, fear memory extinction group. RE, retrieval-extinction group.

Using the miRWalk website to predict the interactions between mRNAs and miRNAs in the F and E/RE groups, 622 miRNAs corresponding to the 10 hub genes were predicted. Then, a differential miRNA analysis was also performed in the F and E/RE groups. Ten miRNAs were significantly differentially expressed in the RE group compared with the F group, of which five were upregulated and five were downregulated in the RE group. Compared with the F group, six miRNAs were significantly differentially expressed, of which three were upregulated and three were downregulated in the E group. The commonly upregulated miRNA between the RE and E groups was mmu-miR-187-3p, and the downregulated miRNA was mmu-miR-200b-3p. According to the predicted relationship between miRNA and mRNA, mmu-miR-187-3p had a regulatory relationship with *Fos* and *Ermn*, and mmu-miR-200b-3p had a regulatory relationship with *Cldn11, Ermn, Fos, Mbp, Plp1*, and *Ugt8a*. Combined with their expression relationship, two target gene relationship pairs corresponding to miRNAs were obtained, namely, mmu-miR-187-3p and Fos, mmu-miR-200b-3p and *Cldn11, Ermn, Mbp, Plp1*, and *Ugt8a*. The TargetScan website was applied to predict the circRNAs regulating mmu-miR-187-3p and mmu-miR-200b-3p, and the predicted circRNAs were intersected with the DECs obtained above. As a result, five downregulated circRNAs corresponding to mmu-miR-187-3p and three upregulated circRNAs corresponding to mmu-miR-200b-3p were obtained (Figures 8A,B). Finally, a ceRNA network composed of eight circRNAs, two miRNAs, and six mRNAs that were co-expressed in memory extinction and retrieval-extinction was constructed (Figure 8C). These findings revealed that ncRNAs, specifically circRNAs, and their effective mechanisms are completely different between fear memory formation and extinction.

Moreover, we predicted eight TFs (PRRX2, LHX8, KLF4, HOXA5, GATA1, EN1, DLX1, and BARHL1) for these eight key circRNAs and displayed the number of binding sites between the TFs and these eight circRNAs (Supplementary Figures S9A–H). Then, these eight TFs were incorporated into the regulatory ceRNA network (Supplementary Figure S10).

## Discussion

Extinction with the reconsolidation window, or retrieval-extinction, has attracted increasing research interest as an underlying technique for targeting the reconsolidation of maladaptive memory with a behavioral intervention (Cahill and Milton, 2019). To the best of our knowledge, this study represents the first comprehensive high-throughput sequencing analysis of circRNA, lncRNA, miRNA, and mRNA expression profiles in the mouse model of F, RE, and E. Dysregulated circRNAs, lncRNAs, miRNAs, and mRNAs showed significant differences in expression between the F/RE/E and control groups, RE/E and F groups, and RE and E groups. We considered these transcripts associated with the processes of memory formation, extinction, and updating. Additionally, we clarified the similarities and differences in signaling pathways, participating molecules, and network mechanisms between fear memory formation and extinction or extinction and updating.

For fear memory formation, the changed mRNAs were mostly involved in some neural-related pathways, such as dopaminergic synapses, glycerophospholipid metabolism, axon guidance, cell adhesion molecules (CAMs), and the RAS signaling pathway, and some BPs, such as central nervous system development, cell proliferation and differentiation, calcium ion response and transport, neurotransmitter metabolic process, and cell-cell signaling and behavior. The effects of all of these pathways on fear memory formation have been reported in many studies (Broussard et al., 2016; Nguyen et al., 2017; Ahmadian-Moghadam et al., 2018; Dorninger et al., 2019; Sun et al., 2019; Asai et al., 2020; Yang et al., 2020), indicating that our experimental system is reliable. For memory extinction in mice, the changed mRNAs were mainly involved in the PI3K-AKT signaling pathway, vascular endothelial growth factor (VEGF) signaling pathway, oxytocin signaling pathway, cGMP-PKG signaling pathway, adipocytokine signaling pathway, neuroactive ligand-receptor interaction, and AMP-activated protein kinase (AMPK) signaling pathway (de Aguiar et al., 2013; Singewald et al., 2015; Wang et al., 2015, 2019; Murphy et al., 2017). Subsequently, the mRNAs that were changed after both fear conditioning and fear extinction were subjected to GO and KEGG enrichment annotations. Ten significant GO-BP terms, 4 significant GO-CC terms, 9 significant GO-MF terms, and 12 significant KEGG pathways were annotated. The regulation of transcription and neuroactive ligand-receptor interaction were both significantly enriched in the processes of fear formation and extinction, suggesting that fear memory extinction was also associated with neural cell activation and new protein synthesis, similar to memory formation. However, the fact that common genes accounted for only 10.78% of the total DEGs indicates that the molecular mechanisms involved in these two memory processes are still quite different. In other words, memory extinction is not simply the formation of new safe memory to compete with the original fear memory, as other important mechanisms are also involved.

Then, the molecular networks of fear memory extinction and updating were delineated and compared further. Predictably, genes related to neuroactive ligand-receptor interactions were also involved in memory updating, similar to many other events occurring in the brain. However, other than this pathway, no other enriched pathways were identified, revealing that memory extinction and retrieval-extinction were two independent processes for reducing fear memory. Moreover, compared with memory extinction, genes that were related to the PI3K-AKT signaling pathway, neuroactive ligand-receptor interaction, calcium signaling pathway, transforming growth factor (TGF-β) signaling pathway, and CAMs might play more important roles in fear memory updating.

Circular RNAs are endogenous transcripts with multiple miRNA response elements, indicating that they potentially interact with the miRNA seed region to alter miRNA activity as a ceRNA (Memczak et al., 2013; Guo et al., 2020). Once hub differentially expressed mRNAs were identified, potential common target miRNAs were predicted by miRWalk and TargetScan tools. Then, the circRNAs corresponding to the target miRNAs were predicted using TargetScan tools. Finally, circRNA-miRNA-mRNA networks in different memory stages were established according to ceRNA theory. Dysfunctional circRNA-miRNA-mRNA regulatory networks represent an essential layer of epigenetic control in central nervous system disorders (Wang et al., 2020). Given the bioinformatics analysis results and ceRNA networks, our study identified 14 circRNAs, 2 miRNAs, and 4 mRNAs involved in memory extinction and retrieval-extinction in mice, which constituted 2 ceRNA networks. Between mice in the memory forming and extinction/retrieval-extinction groups, two circRNA-associated ceRNA networks were established based on eight circRNAs, two miRNAs, and six mRNAs. These molecules have rarely been studied in memory formation, extinction, and retrieval-extinction, but some molecules have been found to be abnormally expressed or to play a role in some neurological diseases. For example, circ_Rims1 is expressed at high levels in rats with vascular dementia (Huang et al., 2020). Notably, miR-200a-3p and miR-200b-3p are downregulated in depressive-like and chronic pain models after 4 weeks of short-term stress (Satyanarayanan et al., 2019). Moreover, miR-143-3p plays an important role in sevoflurane anesthesia-induced cognitive dysfunction (Yu et al., 2021). Several clinical observations have indicated that Adra1d plays an essential role in central nervous system processes (Sadalge et al., 2003; Mishima et al., 2004; Aono et al., 2015). Cldn11, a major component of central nervous system myelin, has been reported to be involved in nerve cell formation processes (Brazert et al., 2020). Studies of cultured oligodendrocytes showed that Plp1 alters the expression of other myelin genes and inhibits the differentiation of oligodendrocyte precursor cells (Karim et al., 2007; Miyamoto et al., 2012). Mbp, the second most abundant protein in central nervous system myelin, is responsible for adhesion of the cytosolic surface of multilayered compact myelin (Boggs, 2006). Moreover, Mbp was discovered to be markedly downregulated in the adult hippocampus along with a considerable reduction in the number of myelinated axons (Xu et al., 2017). In our study, Mbp was expressed at high levels in memory extinction and retrieval-extinction, indicating that Mbp played an important role in reducing fear memory. The roles of other molecules in the development of the nervous system have not yet been reported, and thus our results provide new insights into diseases of the nervous system. Besides, it should be noted that several limitations exist in our study. First of all, behavioral operations that happened in memory formation training, extinction training, and R-E training are different to some extent. When only one control group is compared and analyzed, it cannot be figured out that some molecular changes are caused by the differences in behavioral operations or the memory process itself. Therefore, when the data in this paper are referred for further specific memory process research, it would be better to establish their strictly matched control group for data verification. Second, this study was a preliminary exploratory work, and interactive relationships were predicted mainly *via* some bioinformatics analysis of data from three male mice per group, so in the following, more *in vitro* and *in vivo* experiments and more diverse samples are needed to further support these conclusions.

In summary, by performing whole transcriptome sequencing and multiangle bioinformatics analyses, we investigated the similarities and differences in signaling pathways, key molecules, and circRNA-associated network mechanisms between fear memory formation, and extinction. We also investigated two methods (memory extinction and memory retrieval-extinction) that lead to fear memory decay. Additionally, we identified the key circRNAs, miRNAs, and mRNAs involved in memory updating, which might provide some interesting insights into how retrieval-extinction operations consistently alleviate fear memories.

## Data Availability Statement

The original contributions presented in the study are publicly available. This data can be found here: https://www.ncbi.nlm.nih.gov/geo/, GSE185808.

## Ethics Statement

The animal study was reviewed and approved by Shandong First Medical University.

## Author Contributions

YW designed the study and wrote the manuscript. JS, PL, QZ, XC, XZ, XL, JW, and XY performed all the experiments and analyzed the data. All authors contributed to the article and approved the submitted version.

## Funding

This study was supported by the National Natural Science Foundation (NSFC) (81871059), Academic Promotion Programme of Shandong First Medical University (2019QL016), Shandong Provincial Science and Technology Development Project of Medical and Health (202002050100 and 202002080697), National College Student Innovation and Entrepreneurship Training Program (202110439135), and the Innovation Project of Shandong Academy of Medical Sciences.

## Conflict of Interest

The authors declare that the research was conducted in the absence of any commercial or financial relationships that could be construed as a potential conflict of interest.

## Publisher's Note

All claims expressed in this article are solely those of the authors and do not necessarily represent those of their affiliated organizations, or those of the publisher, the editors and the reviewers. Any product that may be evaluated in this article, or claim that may be made by its manufacturer, is not guaranteed or endorsed by the publisher.
