# Supplementary material for: Identification of the Similarities and Differences of Molecular Networks Associated With Fear Memory Formation, Extinction, and Updating in the Amygdala
Source: Front Mol Neurosci. 2021 Dec 2;14:778170. doi: 10.3389/fnmol.2021.778170 (PMC8675638; doi:10.3389/fnmol.2021.778170)
Supplement: Supplementary file 1 [file Image_1.pdf]

Supplementary figures

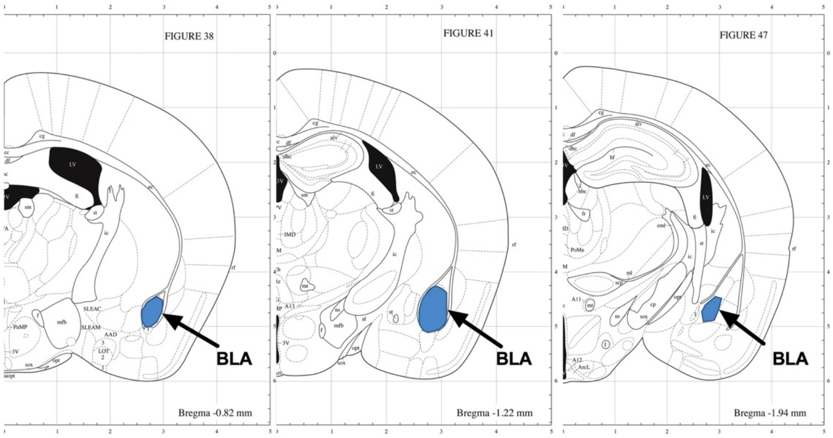

Figure S1. Represented schematic diagram for BLA tissues collection (AP: from -0.82 to -1.94).

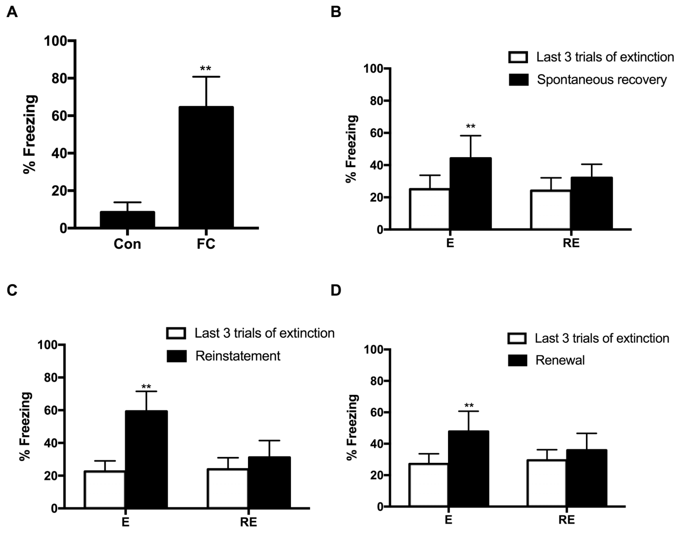

Figure S2. The effects of our different training paradigms on fear memory. A. Fear memory expression after fear conditioning training. Con, control group; FC, fear conditioning training group. **, p <0.01, compared with Con group. B-D. Retrieval-extinction training prevented effectively the spontaneous recovery (B), reinstatement (C) and renewal (D) of cued fear memory from happening. E, extinction training group; RE, retrieval-extinction training group. **, p <0.01,

compared with the means of last 3 trials of extinction. N = 8 per group. All values are presented as the mean ± S.D.

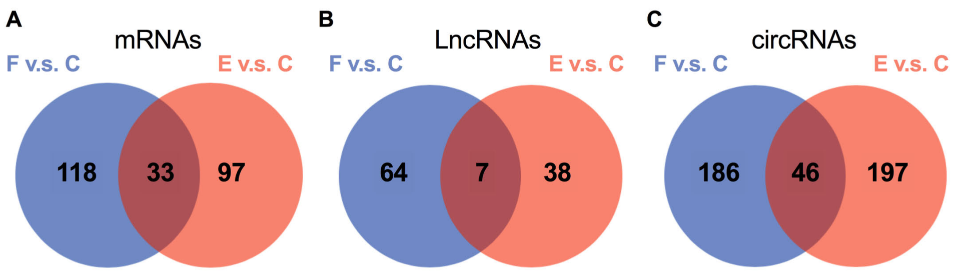

Figure S3. Common changed RNAs after fear memory formation and extinction. Venn diagrams represented the changed mRNAs (A), LncRNAs (B), or circRNAs (C) intersections after fear memory formation and extinction. F, fear memory formation group. E, fear memory extinction group. C, control group.

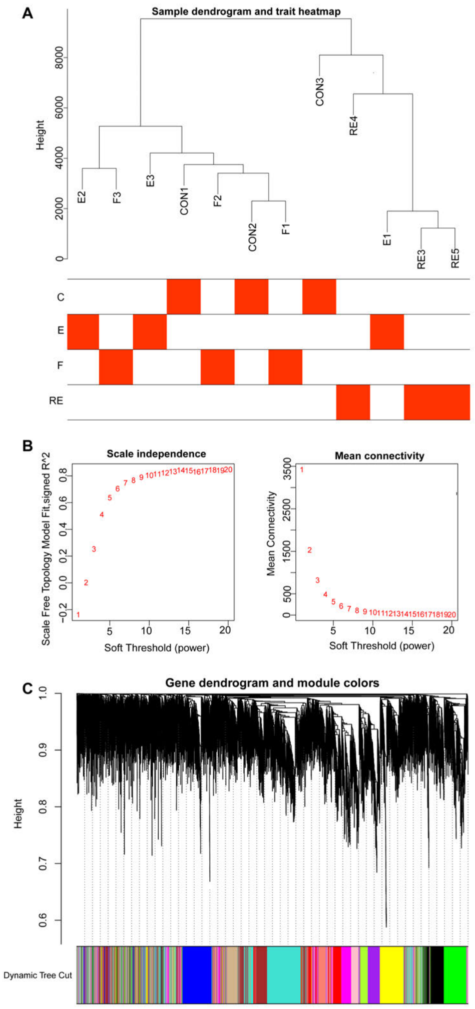

Figure S4. Sequencing results of 12 mice were analyzed by WGCNA. A. Sample clustering was conducted to detect outliers. All samples were located in the clusters and passed cutoff thresholds. B. Soft-thresholding power analysis was applied to get the scale-free fit index of network topology. C. Hierarchical cluster analysis was performed to analyze co-expression clusters with corresponding color assignments. Each color represented a module in the constructed gene co-expression network by WGCNA.

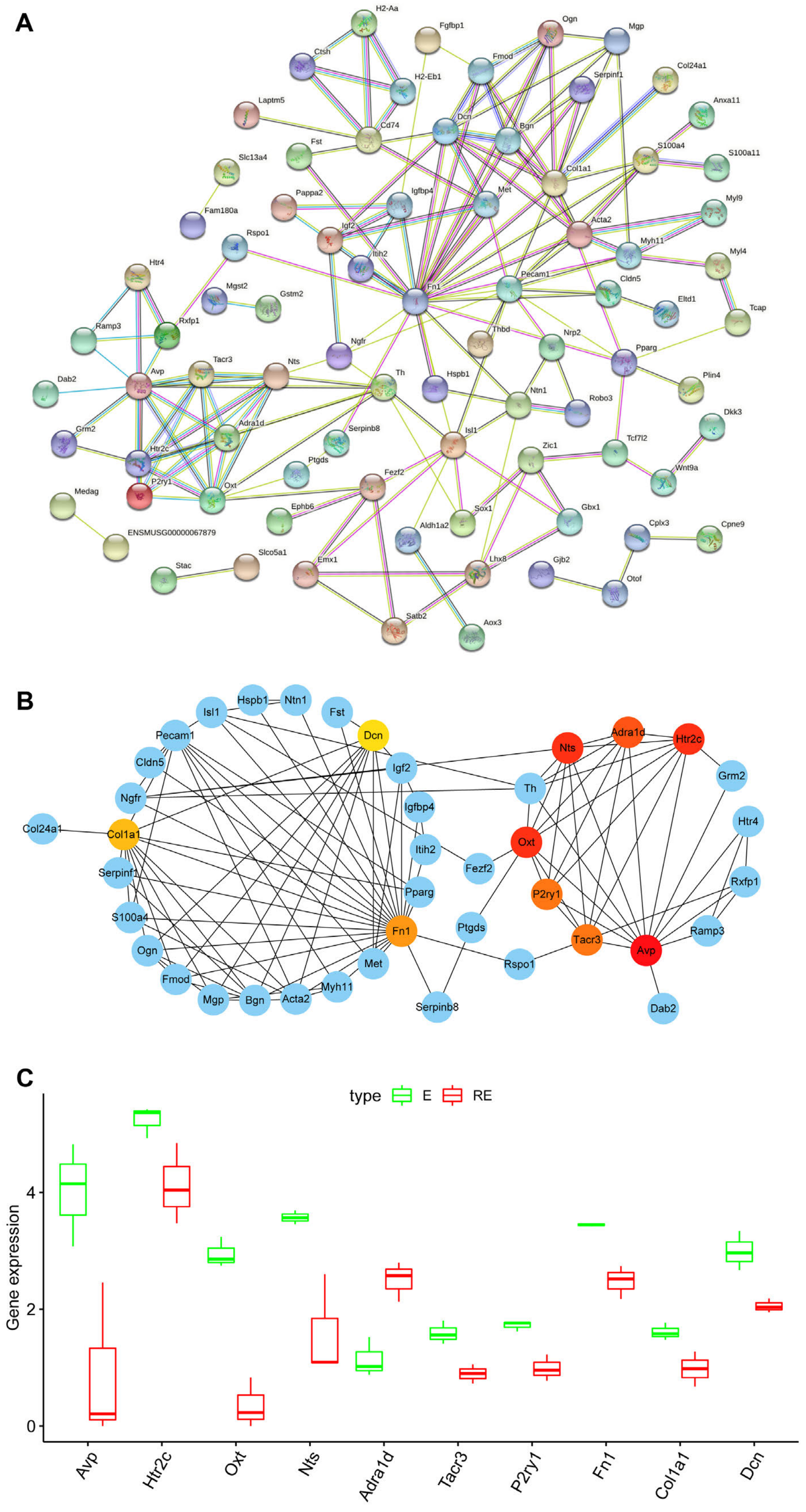

Figure S5. PPI network construction and hub genes selection. A-B.

Visualization of PPI networks among 125 genes in E and RE groups. C. The expression levels of the 10 hub genes in E and RE groups were presented.

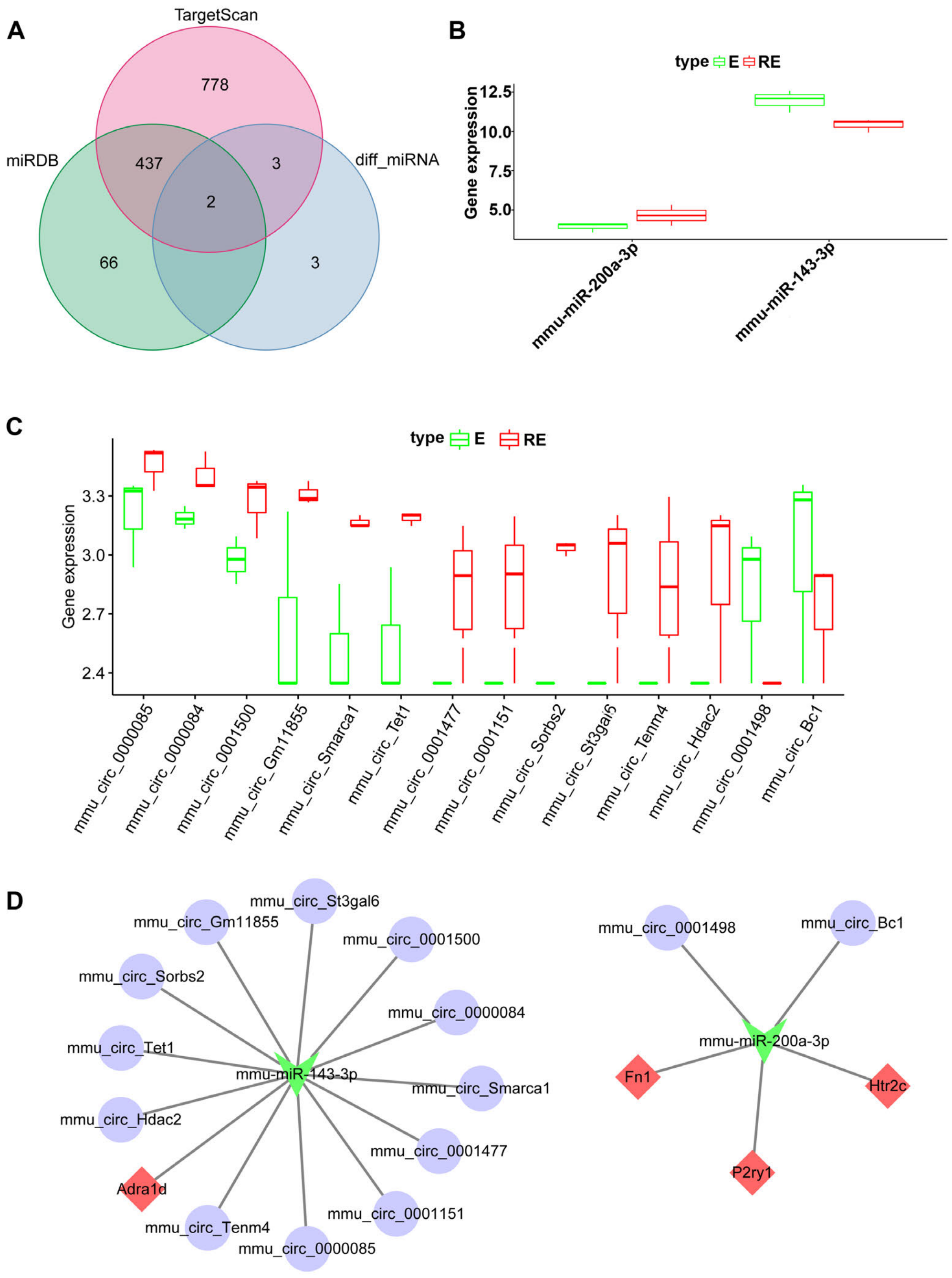

Figure S6. Two circRNA-associated networks were established between E and RE. A. The obtained miRNAs were presented by Venn diagram. B. The expression levels of mmu-miR-200a-3p and mmu-miR-143-3p in E and RE groups were presented. C. The expression levels of 14 circRNAs corresponding to mmu-miR-

200a-3p and mmu-miR-143-3p in E and RE groups were presented. D. Two circRNA-associated networks were established between E and RE. The purple circles represented circRNAs, the green arrowhead represented miRNAs, the red diamonds represented target mRNAs. Network edges represented competitive interactions. E, fear memory extinction group. RE, retrieval-extinction group.

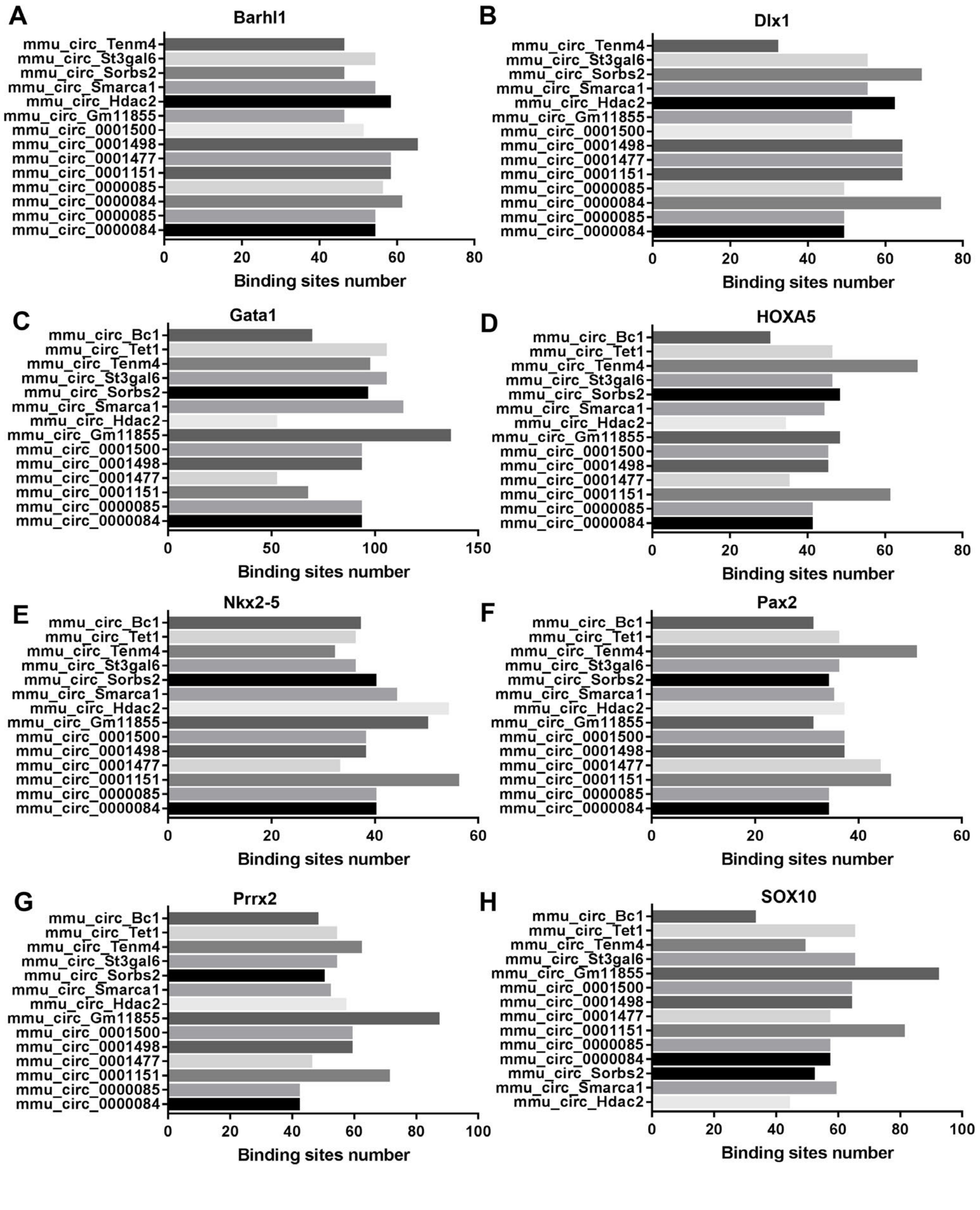

Figure S7. Number of biding sites for each TF to target circRNA that differently changed between RE and E groups. A-H. The binding site number of Barhl1, Dlx1,

Gata1, HOXA5, NIkx2-5, Pax2, Prrx2 and SOX10 corresponding to the 8 circRNAs were presented.

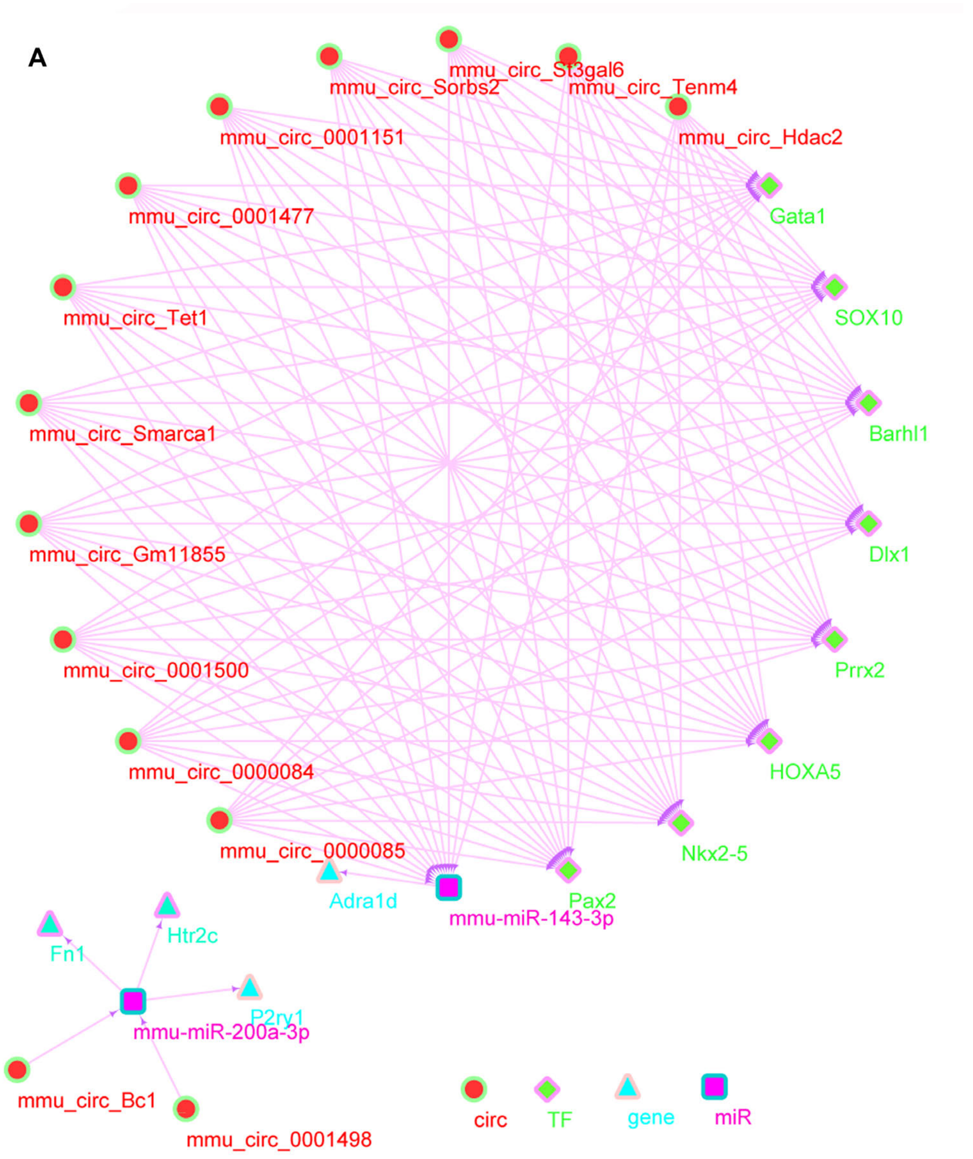

Figure S8. A circRNA-associated ceRNA network was constructed by using circRNAs, miRNAs, mRNAs and TFs in RE and E groups.

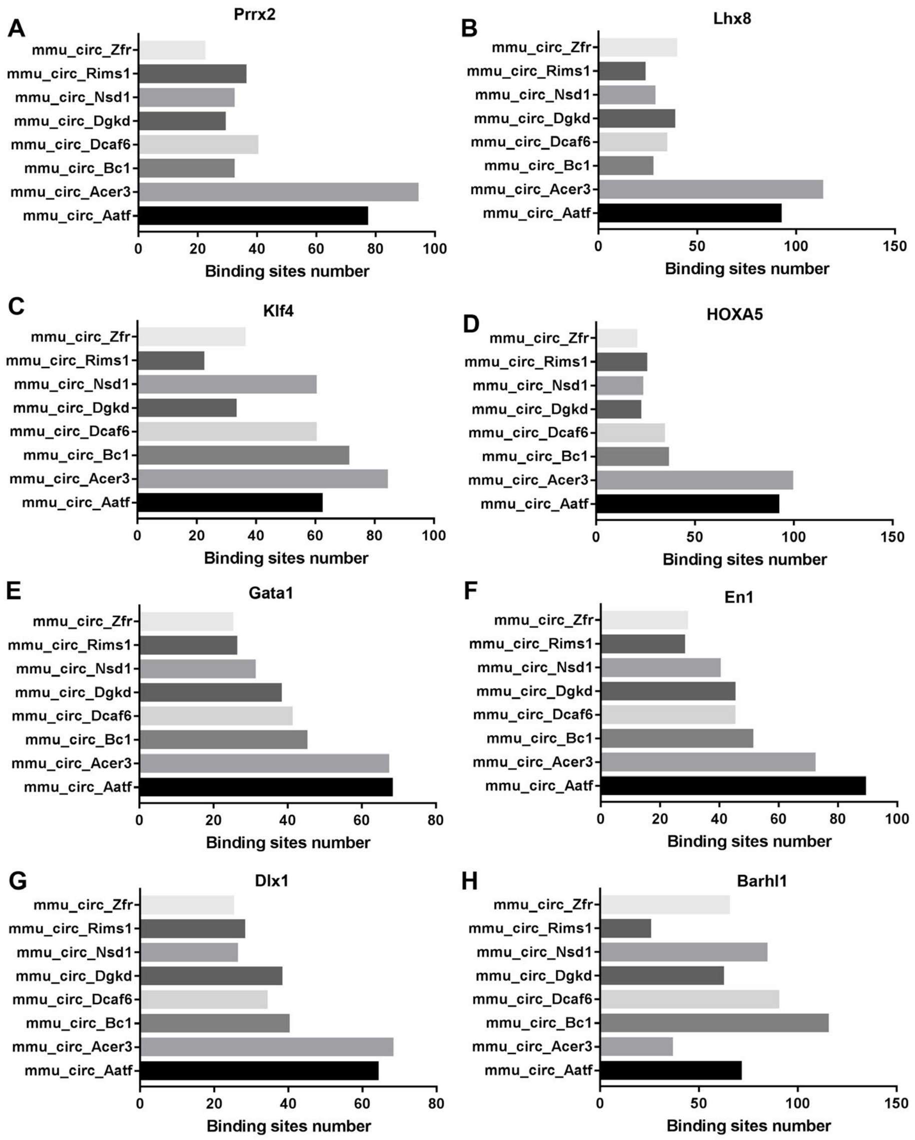

Figure S9. Number of biding sites for each TF to target circRNA that differently changed between F and E/RE groups. A-H. The binding site number of Prrx2, Lhx8, Klf4, HOXA5, Gata1, En1, Dlx1, and Barhl1 corresponding to the 8 circRNAs were presented.

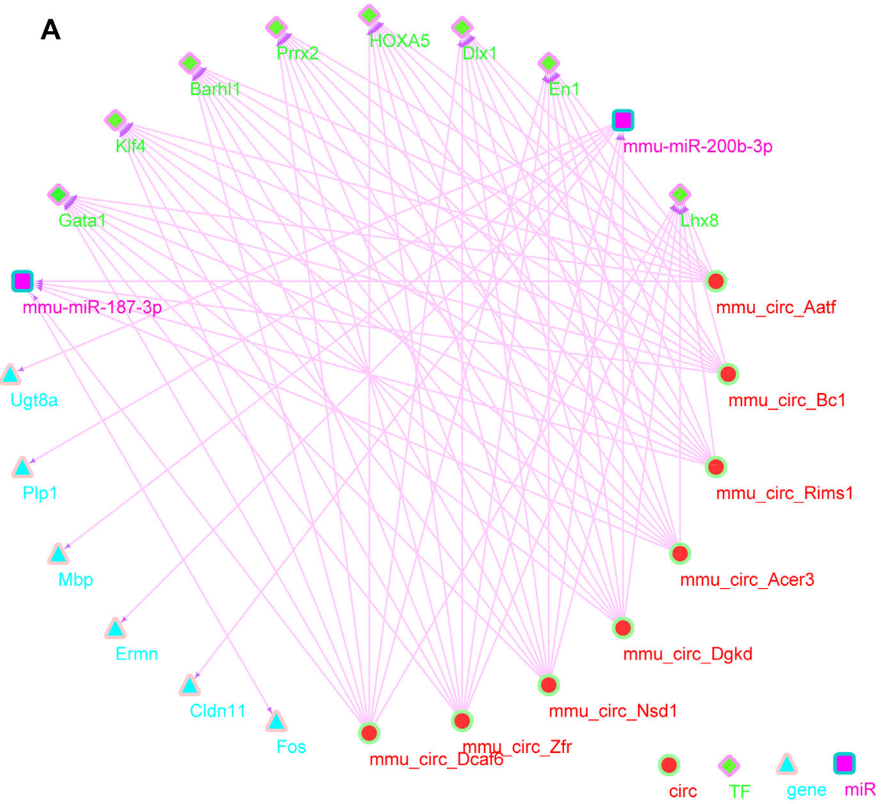

Figure S10. A circRNA-associated ceRNA network was constructed by using circRNAs, miRNAs, mRNAs and TFs in F and E/RE groups.
